# LINC00459 sponging miR-218 to elevate DKK3 inhibits proliferation and invasion in melanoma

**DOI:** 10.1038/s41598-019-55701-x

**Published:** 2019-12-16

**Authors:** Yuhua Yang, Wenxian Xu, Zhuojun Zheng, Zhihai Cao

**Affiliations:** 1 Department of Dermatology, The Third Affiliated Hospital of Soochow University, Changzhou, China; 2 Department of Pathology, The First Affiliated Hospital of Soochow University, Suzhou, China; 3 Department of Hematology, The Third Affiliated Hospital of Soochow University, Changzhou, China; 4 Department of Emergency, The Third Affiliated Hospital of Soochow University, Changzhou, China

**Keywords:** Prognostic markers, Melanoma

## Abstract

The lncRNA biomarkers in melanoma remain to be further explored. The lncRNAs with different expression levels in melanoma tissue were identified by microarray analysis. To investigate the biological functions of target lncRNA, several *in*-*vivo* and *in*-*vitro* studies were performed. Potential mechanisms of competitive endogenous RNAs (ceRNAs) were predicted by using bioinformatics analysis and explored by western blot assay, fluorescence *in situ* hybridization assay, real-time quantitative PCR (RT-qPCR) array, RNA pull-down analysis, AGO2-RIP assay, and dual-luciferase reporter assay. The results demonstrated decreased LINC00459 in melanoma cell lines and tissues. According to the *in-vitro* and *in-vivo* experiments, up-regulated LINC00459 had inhibitory effect on cell proliferation and invasion. Bioinformatics analyses suggested that miR-218 could be a direct target of LINC00459. In addition, miR-218 was proved to be able to directly target the dickkopf-related protein 3 (DKK3) gene. In conclusion, our analysis suggested that the LINC00459 could sponge miR-218 and increase the expression of DKK3 gene, thus inhibiting the invasion and proliferation of melanoma cells, which indicated that the LINC00459 could be an effective biomarker for melanoma and its potential as the therapeutic target.

## Introduction

Skin is the outermost organ of the human body and has absorptive, secretory, immune, and barrier functions. Due to the improvement of diagnostic methods and increased environmental pollution, there has been an increasing trend in the incidence rate and case fatality rate of skin malignant tumors in recent years. Malignant melanoma (MM), evolved or developed from chromatophore nevus or dysplastic nevus, originates from neural crest melanocytes in combination with complex etiological factors, such as solarization, traumatic stimulus, immunosuppression or hereditary variation^1,2^. Melanoma is a kind of severe malignant tumor, ranking third in malignant skin tumors. Currently, with an annual growth rate of 3%-5%, there are approximately 20,000 people suffering from this disease annually^3^. The metastasis and proliferation of MM are very common, and patients with metastasis have poor prognoses, so MM has become one of the diseases that severely endanger human health. MM, as a disease with the highest case fatality rate in skin malignant tumors, is characterized by high-grade malignancy, rapid disease progression, early metastasis to regional lymph nodes and distant organs, limited therapeutic efficiency and insensitivity to chemoradiotherapy. The treatment for MM, benefiting from the progress of immunotherapy, has achieved great improvements, but so far, MM cannot be cured. The majority of patients who are diagnosed with MM can be cured with surgery, but patients with metastasis have a poor prognosis in the late stage, with a survival time generally less than 1 year. The 5-year survival rate is below 15%^4^. There is an increasing trend in the incidence rate of MM^5^.

There are approximately 25,000 protein-coding genes in the human genome, and the majority of genes are transcribed into noncoding RNAs (ncRNAs). ncRNAs can be divided into housekeeping RNAs and regulatory RNAs, with the former including ribosomal RNAs, transfer RNAs and small nuclear RNAs and the latter including micro RNAs and small interfering RNAs. ncRNAs with a length longer than 200 nt are known as long noncoding RNAs (lncRNAs), which are a kind of molecule without protein-coding function that participates in the regulation of gene expression^6^. lncRNA can be transcribed from protein noncoding regions, and they largely consist of ncRNA genes and transcriptional regulatory elements. According to different functions of lncRNAs with respect to tumor initiation and progression, lncRNAs can be divided into cancer-promoting lncRNAs and cancer suppressor lncRNAs, which, when in a relative equilibrium, together participate in cell proliferation and differentiation. If the equilibrium is disturbed, the number and functions of lncRNAs will be affected, which may lead to cell canceration under the action of tumor promoters. Characterized by multiple types, multiple quantities and multiple modes of action, lncRNAs themselves have no protein-coding function, but they can play important roles in genomic imprinting, chromosome modification, gene expression, cell cycle and intranuclear transport^7^ Extensive research on lncRNAs has found that lncRNAs participate in the regulation of expression through a variety of molecular mechanisms. In this study, we identified the functions of a lncRNA and revealed its clinical value by screening lncRNAs with differential expression in melanoma and pigmented nevus tissues using gene chip technology.

## Methods

### Tissue samples

The melanoma and pigmented nevus tissues used in this study were obtained from the First Affiliated Hospital of Soochow University. The samples were achieved in surgeries conducted between February 2014 and November 2018, and were stored at −80 °C. Among all the tissue samples, 21 melanoma samples and 13 pigmented nevus samples were randomly selected for microarray profiling, while the *In-situ* hybridization assay and survival analysis were conducted with the remaining 126 melanoma samples. Informed consents were obtained from all participants. The experimental procedures of human experiments were implemented in strict accordance with Helsinki Declaration, and were approved by the Human Experiment and Ethic Committees of the Third Affiliated Hospital of Soochow University and the First Hospital of Soochow University.

### Microarray profiling

The lncRNAs that were differently expressed in melanoma compared to pigmented nevus tissues (*Padj* < 0.01 or fold change >2.0) were identified by using microarray profiling (Agilent Human LncRNA Microarray, 4*180 K). The experiment was strictly conducted based on the manufacturer’s manual. In brief, the lncRNA microarray chip was hybridized with the cRNAs labeled with Cyanine-3-CTP after cDNA synthesis, labeling and purification. The samples were analyzed by microarray after washing.

### Cell cultures

Melanoma cell lines (A375 and UACC903) and human melanocyte cells (HEM) were purchased from ATCC (Manassas, USA), and the medium of RPMI-1640 with 10% fetal bovine serum was used for cell culture, with the addition of 100 μg/L penicillin-streptomycin.

### Quantitative real-time PCR (qRT-PCR)

Primer synthesis was carried out by Nanjing Genecreate Biological Engineering Co., Ltd, and the sequences were shown in Supplemental Table 1. After extracting the total RNA by TRIzol (Invitrogen, Carlsbad, USA), the primer was amplified with SYBR Green Mix (Promega) through 2^−ΔΔCt^ method. What’s more, the miR-218 expression level was detected by using TaqMan MicroRNA assay kit (Applied Biosystems, Carlsbad, USA). U6 and GAPDH were endogenous control genes for miR-218 and LINC00459, respectively. All the procedures were repeated three times.

### Western blotting experiment

In our study, the total proteins were extracted from cells of logarithmic growth phase by radioimmunoprecipitation assay (RIPA) buffe. The contents were quantified with sodium dodecyl sulfate-polyacrylamide gel electrophoresis, and western blotting analysis was carried out based on the method previously established. GAPDH was applied as the protein loading control. Antibodies against DKK3 (ab2459) and GAPDH (ab181602) were purchased from Abcam (Cambridge, MA, USA).

### Lentivirus and cell transfection

The cDNA lentiviral plasmids (pBLLV-CMV-IRES-ZsGreenLINC00459) for overexpression transfection (Genelily BioTech, Shanghai, China) were transfected with lipofectamine 3000 (Invitrogen). Puromycin at the concentration of 2 μg/mL was used to select the cells that were stably transfected, and qRT-PCR was adopted to determine the transfection efficiency.

### Cell counting kit-8 (CCK8) assay

In our study, the cells were cultured in 96-well plates under the condition of 5% CO_2_ and 37 °C for 5 days. After the addition of CCK8 solution, a microplate reader was applied for cell viability detection and the optical density at 450 nm was measured.

### Flow cytometry

The Annexin V-FITC kit was adopted for the measurement of cell apoptosis^8^. In brief, flow cytometry analysis was conducted in the LINC00459-overexpressed melanoma cells and negative control cells respectively, and CellQuest (BD Biosciences) was adopted for result analysis.

### Transwell assay

Transwell chambers purchased from Corning costar (8-μm pore size; Cambridge, USA) were adopted for evaluation of cell migration and invasion. the lower chamber was added with 20% serum as chemoattractant, and the cells were cultured in the upper chamber for 48 hours. Afterwards, methanol was used for fixation and 0.1% crystal violet was added for cell staining. Due to the gentle abrasion of the cells on the upper surface of the filter, those on the lower surface were counted and photographed under a microscope. All the procedures were conducted in triplicate.

### Wound-healing assay

A375 and UACC903 melanoma cells transfected with LINC00459-overexpressed plasmid and NC plasmid were uniformly inoculated into 6-well plates. A single layer of cells was scraped off from the plate by using a pipette tip to create a gap. Migration of melanoma cells to the gap was monitored under a microscope at a defined time point.

### Fluorescence *in situ* hybridization (FISH)

4% PFA was used to fix the melanoma cells for 15 minutes, and then the cells were permeabilized with 0.5% TritonX-100 at 4 °C for 15 minutes. The cells were cultured with digoxigenin (DIG)-labeled LINC00459 probe or control probe mix for 4 hours at 55 °C. Then, horseradish peroxidase (HRP) conjugated anti-DIG secondary antibody (Jackson, West Grove, PA, USA) was added to detect signals after washing the cells with saline-sodium citrate for 5 minutes (5–6 times). DAPI was applied for nuclear counterstain, and Olympus confocal laser scanning microscope was used for image acquisition.

### *In-situ* hybridization (ISH) assay

Based on a previously described method, the ISH assay was carried out^9^. A locked nucleic acid probe containing the partial complementary sequences to the LINC00459 labeled with digoxigenin antibody (Roche, diluted in 1:1000) was synthesized. The staining intensity was independently assessed by two pathologists that were blinded to this assay.

### Luciferase reporter assay

The pmirGLO-LINC00459-wt and pmirGLO-LINC00459-Mut reporter vectors were composed by the pmirGLO dual-luciferase miRNA target gene expression vectors (Promega) and the LINC00459 cDNA with the predictive miR-218 binding site and its point mutations. The vectors were transfected to the cells together with miR-NC and miR-218. At mean time, the reporter vectors of wild-type and mutant DKK3 3′-UTR were constructed based on the same method. The transfection of the miR-218 or miR-NC together with the vectors into HEK-293FT cells was performed with Lipofectamine 3000 (Invitrogen). 48-hour later, the luciferase reporter trial was strictly performed based on the manual provided by the manufacturer.

### RNA immunoprecipitation

This trial was strictly carried out based on the instructions of the EZMagna RIP Kit (Millipore) provided by the manufacturer. In brief, after cell lysis with RIP lysis buffer, the melanoma cell extract was incubated with anti-argonaute 2 or control anti-IgG antibodies conjugated with magnetic beads for 6 hours at a temperature of 4 °C. The purified RNA was evaluated by using qRT-PCR after removing of the proteins of beads.

### RNA pull-down assay

The A375 cells were transfected with miR or miR-mut at the concentration of 20 nmol/L for 24 hours. The cells were incubated with streptavidin-coated magnetic beads (Life Technologies), and the RNA pull-down assay was conducted in biotin-coupled RNA complex. Finally, the abundance of LINC00459 was calculated based on the qRT-PCR result.

### Animal trials

The BALB/c-nude mice aged four to five weeks were purchased from SLAC (Shanghai, China). The protocol of the animal study was approved by the Animal Care and Use Committees of both the Third Affiliated Hospital of Soochow University and the First Hospital of Soochow University. All mice were raised under appropriate pathogen-free conditions. A375 (5 × 10^6^) cells with different transfection were harvested from culture plates and subcutaneously inoculated into BLAB/C-nude mice. The growth conditions of tumors were monitored and recorded every five days. After one month, the mice were sacrificed, and the tumor tissues were weighed. The volume of tumor tissue was measured in accordance with the formula: volume (cm^3^) = (width^2^ × length)/2

The mice were anesthetizated and had operation with a right lateral flank incision to establish the model of abdominal metastasis. In brief, approximately 100 μL of A375-Luc-vector and A375-Luc-LINC00459 cells (1 × 10^7^) diluted in Hank’s balanced salt solution were injected into the right abdominal cavity. The interactive video information system was adopted for bioluminescence image collection 2 weeks later. We followed the NIH principles of laboratory animal care in order to maintain the animal welfare.

### Statistic methods

We adopted survival analysis to estimate the survival rates, which was carried out by using Kaplan-Meier test and Cox’s regression model. The overall survival time was considered as the time duration between the diagnosis and death of the patients, and PFS was the time period from treatment to disease progression. One-way ANOVA test and the student’s t test were adopted for the comparison of categorical variables and continuous measurements respectively. All the analyses were conducted by using GraphPad Prism 7.0 and SPSS 22.0. P value < 0.05 was defined as the criteria of statistical significance. Numerical data were presented in the form of mean ± SD.

## Result

### Significant decrease of lncRNALINC00459 in melanoma tissues

**Figure 1:**
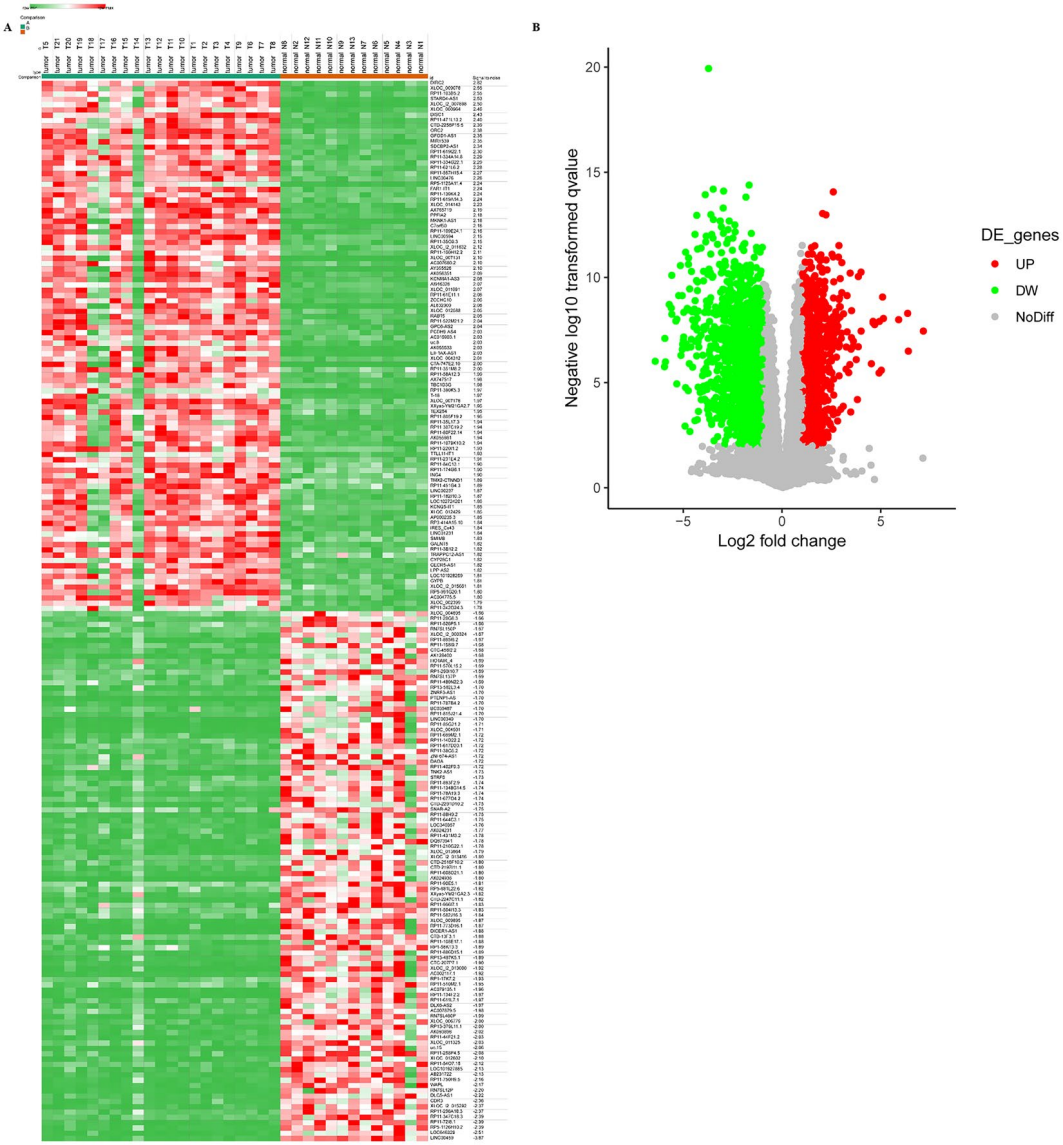
LncRNA LINC00459 is down regulated in melanoma tissues. (**A**) The heat map indicating the 100 lncRNAs that were different in the expression levels between melanoma tissues and pigmented nevus tissues (fold change >2; *P* < 0.01); (**B**) The volcano plot, in which the colored dots representing the lncRNAs (Log_2_(Fold change) > 1 & *P* < 0.01).

Overall 100 lncRNAs with varied expression (Padj < 0.01, Log_2_(fold change) > 1) were observed during screening (Fig. 1A,B). LINC00459 which on chromosome 13q21.31 with 2 exons was significantly decreased in melanoma tissues according to the result of microarray profiling. (Fig. 1A).

### Inhibitory effect of the increased expression of LINC00459 on cell viability and melanoma cell invasion

**Figure 2:**
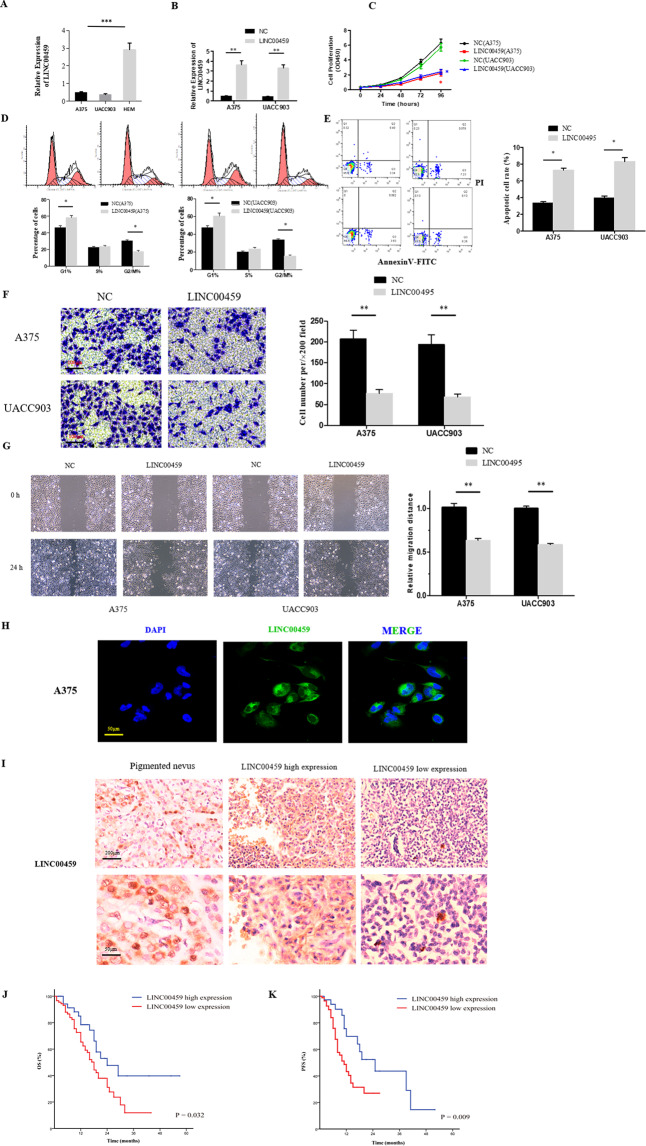
LINC00459 could inhibit *in-vitro* melanoma cell proliferation and predict favorable clinical outcomes. (**A**) The expression of LINC00459 in melanoma cell lines (A375 and UACC903) relative to human melanocytes cells (HEM) detected by RT-qPCR; (**B**) Up-regulation efficiency of the LINC00459 in melanoma cells was investigated by RT-qPCR (**P < 0.01); (**C**) The cell proliferation was determined by CCK8 assay (*P < 0.01); (**D**) FACS analysis suggested declined number of cells from the G2/M phase with overexpressed LINC00459 (*P < 0.01); (**E**) Annexin V assay indicated the overexpression of the LINC00459 could have promotive effect on melanoma cell apoptosis (**P < 0.01); (**F**,**G**) The results of the transwell assay and wound-healing assay of the A375 and UACC903 transfected with Lv-LINC00459 or Lv-NC were presented (**P < 0.01 *vs*. control); (**H**) Based on the fluorescence *in situ* hybridization, majority of the LINC00459 existed in the cytoplasm; (**I**) The expression level of the LINC00459 in melanoma tissues and pigmented nevus tissue was detected by ISH; (**J**,**K**) The patient survival outcomes estimated by Kaplan-Meier Survival curves in melanoma patients with low-level LINC00459 and high-level LINC00459.

The RT-qPCR indicated that the LINC00459 expression in melanoma cell lines was significantly down-regulated comparing to that in human melanocytes cells (HEM) (*P* < 0.05) (Fig. 2A). After transfection of LINC00459, the expression of LINC00459 in A375 and UACC903 cells were up-regulated (Fig. 2B). The cell proliferation (CCK8) in the LINC00459 over-expressed group was reduced compared with the negative control group (Fig. 2C). The cell cycle was inhibited (Fig. 2D) and the cell apoptosis was enhanced in the LINC00459 over-expressed group (Fig. 2E). Both transwell assay and wound-healing assay suggested that increased LINC00459 expression level could significantly weaken the cell invasive capacity (*P* < 0.05) (Fig. 2F,G). The FISH demonstrated the LINC00459 mainly existed in the cytoplasm of the melanoma cell lines (Fig. 2H).

### Favorable impact of the LINC00459 on the survival of patients with melanoma

The survival analysis was performed with overall 126 melanoma tissue samples with complete clinical information. According to the ISH, the expression level of the LINC00459 was decreased in tumor tissues comparing with the pigmented nevus tissues (Fig. 2I). Table 1 showed the patient characteristics at baseline. The patients were divided into two groups based on the expression level of the LINC00459 shown by the results of staining intensity in the ISH assay (score 0–2: low expression; score 3–4: high expression). The median OS in the group of high expression was 24 months, while that in the group of lower expression level was 18 months (Fig. 2J). Based on the univariate log-rank test, the group with high expression achieved a median PFS of 25 months, which was significantly different from that in the low-expression group (11 months) (*P* < 0.01) (Fig. 2K). Multivariate analyses indicated the LINC00459 expression level in melanoma could be an independent risk factor for the OS (HR: 1.752, 95% CI: 1.021–3.219, *P* = 0.036) (Table 2), while lower expression level of the LINC00459 could also lead to less PFS (HR: 2.405, 95% CI: 1.210–4.782, *P* = 0.012) (Table 3). These results indicated a favorable impact of the LINC00459 on the patient survival outcomes of melanoma.

**Table 1:** Clinical characteristics of patients with melanoma.

Characteristic	All patients	LINC00459 (Score 3–4)	LINC00459 (Score 0–2)	*P*-value
Total	126	47	79	
Sex				0.433
Male	62	21	41	
Female	64	26	38	
Age(years)				0.327
<60	82	31	51	
≥60	44	16	28	
BMI				
<24	97	38	59	0.42
≥24	29	9	20	
ECOG score				
0–2	112	42	70	0.896
3–4	14	5	9	
Weight loss				
<5%	115	43	72	0.946
≥5%	11	4	7	
TNM stage				
I–II	97	34	63	0.340
III–IV	39	13	16	

Abbreviations: BMI, Body Mass Index; ECOG, Eastern Cooperative Oncology Group.

**Table 2:** Univariate and multivariate analysis of melanoma patients on overall survival.

Variable	Univariate analysis		Multivariate analysis	
	HR (95% CI)	*P*	HR (95% CI)	*P*
Sex (Male *vs*. Female)	1.033 (0.747–1.725)	0.153		
Age (≥60 *vs*. <60 years)	1.192 (0.576–1.879)	0.223		
BMI (≥24 *vs*. <24)	0.954 (0.638–1.266)	0.104		
ECOG score (3–4 *vs*. 0–2)	1.524 (1.028–2.495)	**0.042**	1.321 (0.875–2.617)	0.381
Weight loss (≥5% *vs*. <5%)	1.326 (0.682–2.713)	0.404		
TNM stage (III-IV *vs*. I-II)	2.241 (1.247–3.078)	**0.021**	2.137 (1.201–2.975)	**0.027**
LINC00459 expression (low *vs*. high)	1.853 (1.031–3.329)	**0.039**	1.752 (1.021–3.219)	**0.036**

Abbreviation: BMI, Body Mass Index; ECOG, Eastern Cooperative Oncology Group.

**Table 3:** Univariate and multivariate analysis of melanoma patients on progression free survival.

Variable	Univariate analysis		Multivariate analysis	
	HR (95% CI)	*P*	HR (95% CI)	*P*
Sex (Male *vs*. Female)	0.925 (0.442–1.925)	0.391		
Age (≥60 *vs*. <60 years)	1.041 (0.734–2.081)	0.553		
BMI (≥24 *vs*. <24)	0.892 (0.638–1.666)	0.367		
ECOG score (3–4 *vs*. 0–2)	1.315 (0.728–2.195)	0.564		
Weight loss (≥5% *vs*. <5%)	1.221 (0.738–1.913)	0.478		
TNM stage (III-IV *vs*. I–II)	2.284 (0.955–5.464)	0.064		
LINC00459 expression (low *vs*. high)	2.405 (1.210–4.782)	**0.012**	2.405 (1.210–4.782)	**0.012**

Abbreviation: BMI, Body Mass Index; ECOG, Eastern Cooperative Oncology Group.

### *In-vivo* verification of the impact of LINC00459

**Figure 3:**
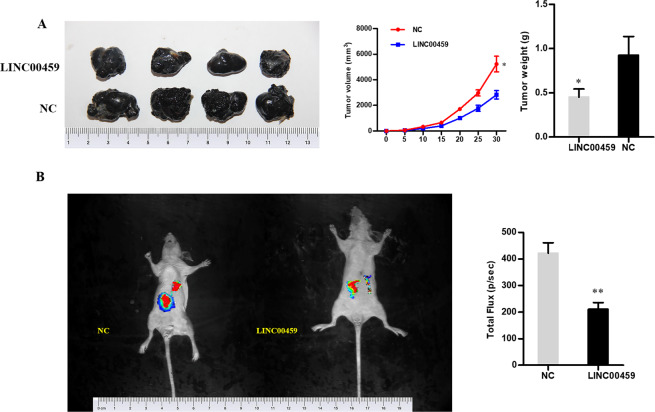
The effect of the LINC00459 on *in-vivo* proliferation and metastasis. (**A**) Volume and weight of the tumors excised from the nude mice injected with A375 cells and the overexpressed LINC00459 or overexpressed NC; (**B**) The bioluminescent imaging of the mice was collected by IVIS bioluminescence imaging system after transplantation of control cells and luciferase expressing LINC00459 A375 cells (**P < 0.01).

The A375 cells with overexpressed LINC00459 and corresponding negative control cells were administrated to BALB/c-nude mice via subcutaneous injection to verify the impact of LINC00459 *in vivo*. Smaller tumor volume and lower tumor weight were observed in the mice with overexpressed LINC00459 (Fig. 3A). The results of the abdominal metastasis model revealed that overexpressed LINC00459 had significant inhibitory effect on cell invasion *in vivo* (*P* < 0.05) (Fig. 3B).

### LINC00459 as an ceRNA of miR-218 and the relevant regulation effect on the target gene of miR-218 (DKK3)

**Figure 4:**
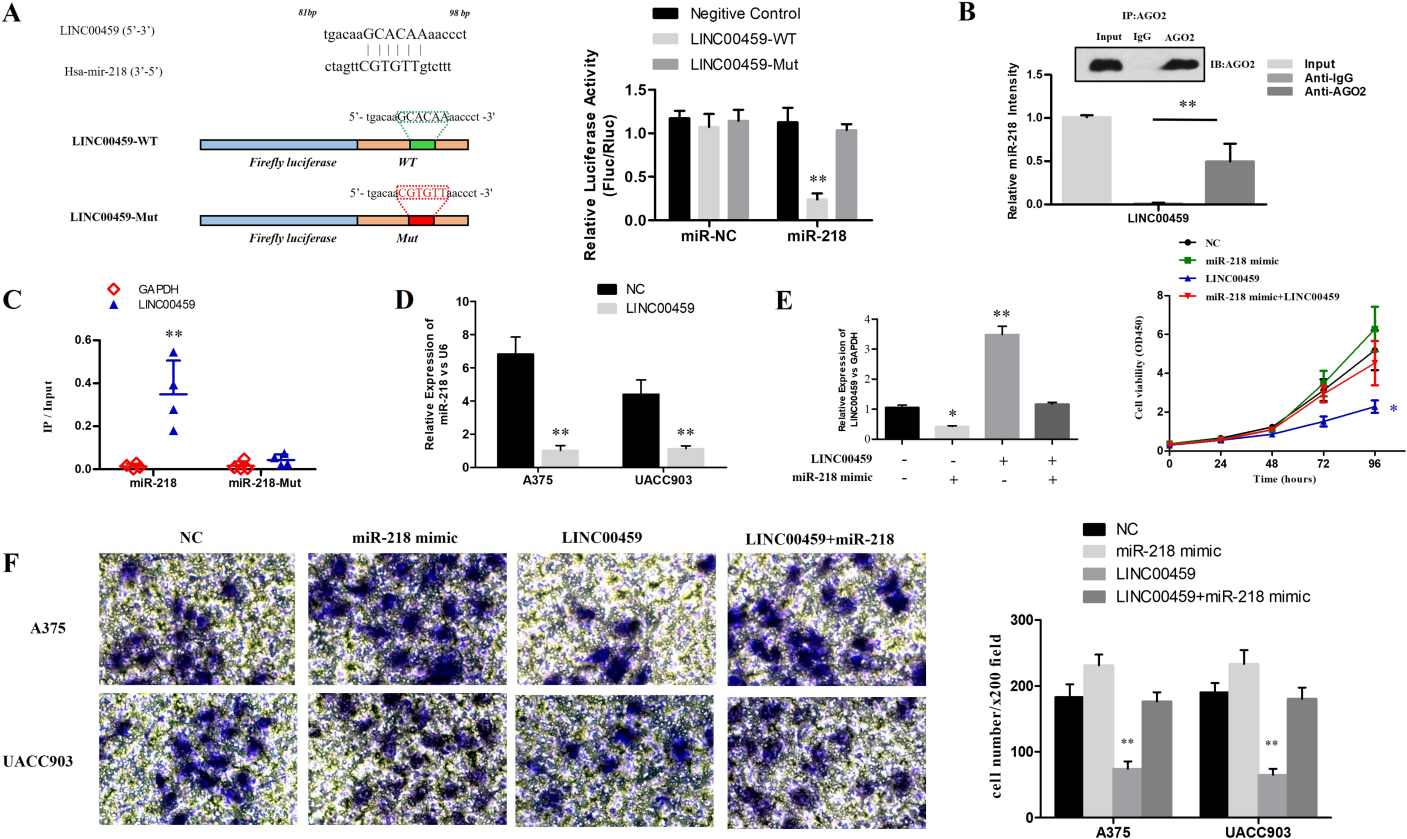
The targeted regulation of the LINC00459 on miR-218. (**A**) The upper diagram showed the schematic figure of the predictive miR-218 binding sites of the LINC00459, and the lower diagram presented the sequences of LINC00459 and LINC00459. The histogram presented the luciferase activities in A375 cells (**P < 0.01); (**B**) Anti-AGO2 RIP assays showed that LINC00459 and miR-218 were more abundant in anti-AGO2 comparing with the anti-IgG immunoprecipitates (**P < 0.01); (**C**) The scatter plot showed that the content of LINC00459 was higher in the miR-218-wt than in miR-218-mut with mutant binding sites of LINC00459 (**P < 0.01); (**D**) The expression of miR-218 in melanoma cells with overexpressed LINC00459 relative to the cells with overexpressed-NC were presented; (**E**) The expression of LINC00459 and cell proliferation after transfection of NC, miR-218 mimic Lv-LINC00459 and miR-218 mimic + Lv-LINC00459 were evaluated by RT-qPCR and CCK-8 assay (*P < 0.05, **P < 0.01); (**F**) The invasion of melanoma cells after transfection of NC, miR-218 mimic, Lv-LINC00459 and miR-218 mimic + Lv-LINC00459 were detected by transwell assay (*P < 0.05, **P < 0.01).

The expression of lncRNA can be decreased by absorbing a variety of miRNAs, thus leading to reduced regulatory effect on the target mRNA. Based on the bioinformatics (miRcode http://www.mircode.org/), the reverse complementary recognition sequence of LINC00459 (target miRNA) was predicted, and the most relevant target miRNA has been screened out in the RNA pull-down analysis. According to our analysis, comparing to other miRNAs, the LINC00459 was more abundant in the miR-218 (Supplemental Fig. 1A). Therefore, miR-218 was selected for further research. Luciferase reporter assay of the pmirGLO-LINC00459-wt reporter vector indicated that the cells transfected with the reporter vector and miR-218 had significant lower luciferase activity compared to those transfected with miR-NC. At mean time, the assay of pmirGLO-LINC00459-mut reporter vector showed comparable luciferase activity in the two groups (Fig. 4A). The anti-Ago2 immunoprecipitates containing miRNAs and their interacting RNA-components was used in the RIP assay^10–12^. The assay indicated that, comparing with the anti-IgG immunoprecipitates, the LINC00459 and miR-218 were more abundant in AGO2 (Fig. 4B). In addition, according to the RNA pull-down analysis, the content of the LINC00459 was higher in the miR-218-wt than in the miR-218-mut with mutant binding site of the LINC00459 (Fig. 4C). What’s more, the RT-qPCR demonstrated that the increasing LINC00459 level could inhibit the expression of miR-218 in melanoma cells (Fig. 4D). Additionally, the rescue experiment suggested that the suppression of cell proliferation (Fig. 4E) and invasion (Fig. 4F) that caused by overexpression of LINC00459 could be reversed by the transfection of miR-218 mimic in melanoma cells to a great extent. Transfection of miR-218 mimic in melanoma cells alone could enhance the proliferation and invasion ability in melanoma cells, but the difference was not statistically significant comparing to control group.

**Figure 5:**
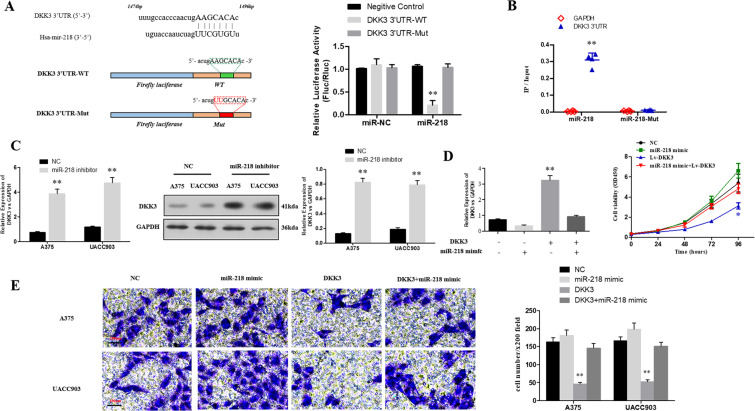
DKK3 could be targeted and regulated by miR-218. (**A**) The predicted miR-218 binding sites within the 3′UTR of DKK3 were presented in the schematic diagram. The sequences of wild-type and mutant 3′UTR of DKK3 were also presented. The luciferase activity in A375 cells was detected by using luciferase reporter gene assays (**P < 0.01); (**B**) Biotinylated miR-218 or its mutant (miR-218-mut) was transfected into A375 cells. The RNA levels of the 3′UTR of DKK3 and GAPDH were quantitatively analyzed by the RT-qPCR. The relative ratios of the input of IP were presented in the scatter plot (**P < 0.01); (**C**) The relative expression of mRNA and protein of DKK3 in melanoma cells with miR-218 inhibitor and NC were presented; (**D**) The expression of DKK3 and cell proliferation after transfection of NC, miR-218 mimic Lv-DKK3 and miR-218 mimic + Lv-DKK3 were determined by RT-qPCR and CCK-8 assay (**P < 0.01); (**E**) The invasion of melanoma cells after transfection of NC, miR-218 mimic, Lv-DKK3 and miR-218 mimic + Lv-DKK3 was evaluated by transwell assay (**P < 0.01).

The binding sites in several genes’ 3′-UTR with miR-218 were predicted by the bioinformatics (Targetscan 7.2 http://www.targetscan.org/vert_72/; miRDB http://www.mirdb.org/; miRTarBase http://mirtarbase.mbc.nctu.edu.tw/php/index.php). Dickkopf-related protein 3 (DKK3) was considered as the target gene based on the screening of RT-qPCR (Supplemental Fig. 1B). The luciferase assay showed that the 3′-UTR of wild-type DKK3 had the ability to significantly inhibit the luciferase activity of the cells transfected with miR-218, while showed no impact on the cells transfected with miR-NC. What’s more, 3′-UTR of mutant-type DKK3 did not significantly influence the luciferase activity of the cells transfected with miR-218 (Fig. 5A). It was confirmed in the RNA pull-down assay that the content of DKK3 3′-UTR was higher in the wild-type miR-218 compared with the mutant-type miR-218 with mutant DKK3 3′-UTR binding site (Fig. 5B). The RT-qPCR and western-blotting assay suggested that the level of miR-218 could have impact on the mRNA and protein expression in DKK3 (Fig. 5C). Moreover, the up-regulation of miR-218 in melanoma cells had reversal effect on the inhibition of cell viability (Fig. 5D), invasion and migration (Fig. 5E) induced by DKK3 overexpression.

### Verification of regulation of LINC00459/miR-218/DKK3 pathway on melanoma cell *in vivo*

**Figure 6:**
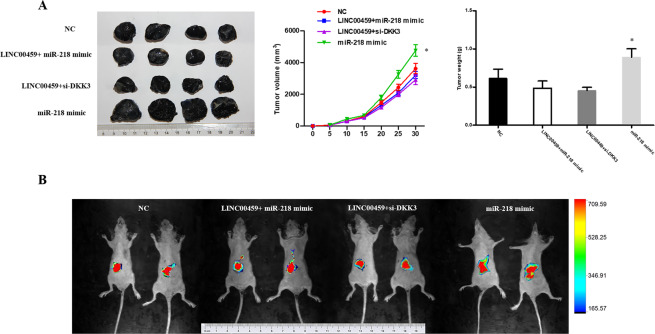
The validation of LINC00459/miR-218/DKK3 pathway on proliferation *in vivo*. (**A**) A375 cells that transfected with NC, Lv-LINC00459 + miR-218, Lv-LINC00459 + Lv-siDKK3 and miR-218 mimic were injected to the back of BALB/c-nude mice subcutaneously. Tumor volume was measured every 5 days. Mice were sacrificed at one month later. Tumors were excised and tumor weight were then analyzed. *P < 0.05. (**B**) The bioluminescent imaging of the mice was collected by IVIS bioluminescence imaging system after transplantation of luciferase expressing A375 cells transfected with NC, Lv-LINC00459 + miR-218, Lv-LINC00459 + Lv-siDKK3 and miR-218 mimic.

The A375 cells that transfected with NC, Lv-LINC00459 + miR-218, Lv-LINC00459 + Lv-siDKK3 and miR-218 mimic were injected to the back of BALB/c-nude mice subcutaneously. The tumors with bigger volume and heavier weight could be observed in miR-218 mimic group than other groups. The difference was not statistically significant among other 3 groups which was in accordance with the results *in vitro* (Fig. 6A). The similar results were observed in abdominal metastasis model as well (Fig. 6B).

## Discussion

Currently, there is no unified standard for the classification and denomination of lncRNAs^13^. However, lncRNAs can be divided into 5 types according to their localization in the genome with respect to protein-coding genes, as follows^14^: ① homomeric lncRNA, which means that the lncRNA overlaps with one or more exons of another transcript on the same DNA strand; ② antisense lncRNA, which means that the lncRNA is transcribed from one or more exons of the neighboring protein-coding gene repeatedly in the opposite direction; ③ intergenic lncRNA, which means that the lncRNA is transcribed from the region between 2 genes; ④ intragenic lncRNA, which means that the lncRNA is completely transcribed from an intron of another transcript; and ⑤ bidirectional lncRNA, which means that the lncRNA is located near the transcription start site of the protein-coding transcript on the complementary strand (<1000 bp). However, with the continuous studies on lncRNA mechanisms, the powerful and extensive biological functions of lncRNAs have been shown to play an indispensable role in organisms. Studies have shown that lncRNAs, mainly at the transcriptional/posttranscriptional level and the translational/posttranslational level of epigenetic inheritance, play the roles of signal molecules, decoy molecules, guide molecules and scaffold molecules to achieve gene expression^15^ and regulate DNA methylation, histone modification and chromatin remodeling to silence or activate genes^16^ and exert their biological functions. ① lncRNAs can protect protein-coding genes in many ways. ② lncRNAs can participate in chromatin modification, X-chromosome silencing, genetic imprinting, transcriptional interference and activation, intranuclear transport and many other important physiological and biochemical regulatory processes. ③ lncRNAs with complex structural and functional areas play the role of regulatory factors, such as promoters and terminators. ④ lncRNAs can be transcribed from the conserved regions of evolutionarily related genes. ⑤ lncRNA can mediate sequence-specific regulation^17^.

At present, there are few correlational studies on lncRNA and MM at the genetic level. Through the combination with polycomb repressive complex 1 (PRC1), HOTAIR can achieve the trimethylation of the 27^th^ lysine of histone H3 and regulate Wnt/β link protein, phosphatidylinositol 3-kinase and other signaling pathways through gene silencing to promote tumor invasion and metastasis, avoid growth inhibition and resist cell apoptosis^18^. Furthermore, HOTAIR can combine with histone demethylase 1 to inhibit proteins from forming a complex and regulate the transcriptional activation of target genes^18^. Therefore, HOTAIR can influence tumor initiation and progression through epigenetics by forming a complex with PRC2 or histone demethylase 1. Based on the detection of MM primary lesions and metastatic lesions using RT-PCR, Tang *et al*.^19^ found that HOTAIR is overexpressed in MM, with the highest expression in lymph node tissues that have been transferred and disseminated, which shows that HOTAIR is closely related to the development and metastasis of MM^20,21^. lncRNA-SPRY4-IT1 was initially discovered in the sequencing of adipose tissues. As shown in previous studies, the low expression of SPRY4-IT1 can promote the accumulation of fat-soluble substances, cause cells to produce cytotoxic substances and induce cell apoptosis, which can affect the metabolic activity of cells and regulate cell death, invasion and migration^22^. The expression of SPRY4-IT1 is increased in malignant tumors. SPRY4-IT1 regulates the proliferation, metastasis and apoptosis of tumor cells mainly through chromosome methylation, cell cycle protein regulation and expression pathways related to proteolipid proteins to play a role in epithelial-mesenchymal transition. Khaitan *et al*.^23^ found that SPRY4-IT1 primarily aggregates in the cytoplasm of MM cells. A reduction in its expression leads to a growth defect in MM cells and further decreases the invasion and increases the apoptosis rate. Luo *et al*.^24^ found that H19, with its function as a cancer suppressor gene, can combine with histone-lysine N-transmethylase to inhibit the expression of epithelial cadherin by directly inhibiting the transcription of epithelial cadherin and indirectly stimulating the Wnt/β signaling pathway to promote the epithelial-mesenchymal transition, invasion and migration of tumor cells. So far, there has been no report about the significant correlation between MM and the H19 gene. However, Mazar *et al*.^22^ found that H19 polymorphism may influence the occurrence and development of MM. MALAT1 can change the splicing of pre-RNAs. This process affects the level of posttranscriptional modification by regulating the phosphorylation level of splicing factor, a member of the serine/arginine-enriched protein family^25^. The different expression levels for MALAT1 can influence the expression of Myb-related protein B (B-MYB), a carcinogenic transcription factor involved in the progression to G2/M stage. MALAT1 regulates mitosis by controlling the expression level of the B-MYB transcription factor and thus regulates the cell cycle. The low expression of MALAT1 can interrupt cell mitosis, activate the p53 gene and impair E2F1 activity and cell proliferation to inhibit the division and proliferation of cancer cells^26^. It has been discovered through *in vitro* experiments with MM cells that the metastasis capacity of MM can be reduced by decreasing the expression of MALAT1. In contrast, increased MALAT1 expression can increase the metastasis capacity of MM^27^. It is thus clear that the expression level of MALAT-1 is closely related to the metastasis degree of MM. On the basis of RT-PCR results, it can be observed that UCA1 has different expression levels in the early stage and late stage of MM. Moreover, it has been discovered through an *in vitro* experiment that the metastasis capacity of MM can be increased greatly by increasing the expression of UCA1^28^. BANCR, induced by the BRAF gene, is a new and potential lncRNA product that participates in the proliferation of MM cells. As shown in RT-PCR results, BANCR is overexpressed in MM tissues and cell lines. Its expression level is positively correlated with neoplasm staging, and no significant correlation was observed with the gender and age of patients and the location and size of tumors^29^. The low expression of BANCR can inhibit the proliferation of MM cells *in vitro* and *in vivo*, which is related to its inhibitory effect on mitogen-activated protein kinase (MAPK) because it can inhibit the growth of tumors by inhibiting the survival and proliferation of cells^30^. Hence, the BANCR and MAPK pathways can regulate cell proliferation synergistically. Flockhart *et al*.^31^ showed that low BANCR expression can effectively control the proliferation and metastasis of MM cells. Patients with high expression of BANCR have a relatively short survival time, which indicates that the expression level of BANCR may become a predictive index for poor clinical prognosis of MM^31^. It was found in 103 cases of MM tissues and 5 MM cell lines with clinical staging that the expression of BANCR was distinctly upregulated. The infiltration of melanoma cells can be inhibited *in vitro* and *in vivo* by knocking out BANCR in sk-mel-5 cells.

In this study, we found that the expression of LINC00459 in melanoma tissues is much lower than that in pigmented nevus tissues. We also discovered that the expression of LINC00459 in melanoma cell lines is also lower than that in normal melanophores. In melanoma cell lines A375 and UACC903, the exogenous expression of LINC00459 is increased, the cell viability is reduced, the proportion of cells in the G2/M stage is decreased, the apoptosis rate is increased, and the invasion and migration capacities of cells are weakened. *In vivo*, we found that the tumor formation volume and tumor mass of naked mice injected with melanoma cells overexpressing LINC00459 were much lower than those of mice in the blank control group. *In vivo* imaging results showed that, compared with the cells in the control group, the melanoma cells overexpressing LINC00459 had poorer invasion capacity in the abdominal cavity of mice. Moreover, we performed a retrospective analysis on the tumor tissue samples of 126 patients diagnosed with melanoma. In addition, we divided the patients with 0–2 scores as the low LINC00459 expression group and those with 3–4 scores as the high LINC00459 expression group based on the scores (0–4) for the expression level determined by *in situ* hybridization (ISH). The results of multiple-factor analysis show that the expression of LINC00459 is an independent risk factor that influences the overall survival and progression-free survival of patients and is not affected by disease staging. By comparing the overall survival (OS) and progression-free survival (PFS) of the two groups of patients, we observed that the median OS and PFS of patients in the high LINC00459 expression group are much higher than those of patients in the low LINC00459 expression group, which shows that LINC00459 has promising clinical value for the prognosis of melanoma. To better understand the mechanism that LINC00459 impacts progression of melanoma, we further identified and validated LINC00459/miR-218/DKK3 pathway that regulated the proliferation and invasion ability of melanoma cells. LINC00459 may act as an endogenous competitive RNA to sponge miR-218 and thus moderate the effector DKK3 to regulate the proliferation and migration of melanoma. miR-218 usually was regarded as a tumor suppressor in several cancers, such as pancreatic cancer, prostate cancer, endometrial cancer, breast cancer and so on^32–35^. It is seldom reported as an oncogene except in non-small cell lung cancer^36^. Here we have new findings. It actually acts as a “bad guy” in melanoma. While DKK3 always be a “good man” in cancers^37–39^ which is no exception in this study. LINC00459/miR-218/DKK3 pathway has enriched the mechanism of development of melanoma to some extent. Here we did not perform loss-of-function assays as the expression of LINC00459 in melanoma was relatively low. Further knock down of LINC00459 may cause little positive biological effect to be observed which could be partly reflected in above rescue experiments. There also were some limitations of this study. Activation of classical signaling pathways need to be verified. Clinical sample size needs to be enlarged.

In conclusion, our research results indicate that the LINC00459/miR-218/DKK3 pathway may be a novel therapeutic target and biomarker in melanoma. So far, there is a lack of research on LINC00459. However, an in-depth study of its biological functions and regulatory mechanisms can help us better understand the pathogenic mechanism of melanoma and further provide better guidance for clinical treatment, which will be the main direction of our future studies.

## Supplementary information

Supplementary information

## Data Availability

The datasets used and analyzed during the current study are available from the corresponding author on reasonable request.

## References

[1] Ligtenberg MA (2018). Self-Delivering RNAi Targeting PD-1 Improves Tumor-Specific T Cell Functionality for Adoptive Cell Therapy of Malignant Melanoma. Mol Ther.

[2] Xing Y (2017). CANT1 lncRNA Triggers Efficient Therapeutic Efficacy by Correcting Aberrant lncing Cascade in Malignant Uveal Melanoma. Mol Ther.

[3] Li CH, Chen Y (2013). Targeting long non-coding RNAs in cancers: progress and prospects. Int J Biochem Cell Biol.

[4] Siurala M (2016). Adenoviral Delivery of Tumor Necrosis Factor-alpha and Interleukin-2 Enables Successful Adoptive Cell Therapy of Immunosuppressive Melanoma. Mol Ther.

[5] McCart JA (2002). Development of a melanoma-specific adenovirus. Mol Ther.

[6] Xie M (2018). Potential Regulatory Roles of MicroRNAs and Long Noncoding RNAs in Anticancer Therapies. Mol Ther Nucleic Acids.

[7] Perkel JM (2013). Visiting “noncodarnia”. Biotechniques.

[8] Koblížek Miroslav, Lebedeva Anastasia, Fišer Karel (2018). flowIO: Flow cytometry standard conformance testing, editing, and export tool. Cytometry Part A.

[9] Dong J (2017). HMGA2-FOXL2 Axis Regulates Metastases and Epithelial-to-Mesenchymal Transition of Chemoresistant Gastric Cancer. Clinical cancer research: an official journal of the American Association for Cancer Research.

[10] King VM, Borchert GM (2017). MicroRNA Expression: Protein Participants in MicroRNA Regulation. Methods in molecular biology.

[11] Long JM (2016). Genome-scale mRNA and small RNA transcriptomic insights into initiation of citrus apomixis. Journal of experimental botany.

[12] Zhang W, Le TD, Liu L, Zhou ZH, Li J (2016). Predicting miRNA Targets by Integrating Gene Regulatory Knowledge with Expression Profiles. PloS one.

[13] Gibb EA, Brown CJ, Lam WL (2011). The functional role of long non-coding RNA in human carcinomas. Mol Cancer.

[14] Hauptman N, Glavac D (2013). Long non-coding RNA in cancer. Int J Mol Sci.

[15] Schmitt AM, Chang HY (2016). Long Noncoding RNAs in Cancer Pathways. Cancer Cell.

[16] Chen LL, Carmichael GG (2010). Decoding the function of nuclear long non-coding RNAs. Curr Opin Cell Biol.

[17] Brosnan CA, Voinnet O (2009). The long and the short of noncoding RNAs. Curr Opin Cell Biol.

[18] Gupta RA (2010). Long non-coding RNA HOTAIR reprograms chromatin state to promote cancer metastasis. Nature.

[19] Tang L, Zhang W, Su B, Yu B (2013). Long noncoding RNA HOTAIR is associated with motility, invasion, and metastatic potential of metastatic melanoma. Biomed Res Int.

[20] Cantile M (2017). HOTAIR role in melanoma progression and its identification in the blood of patients with advanced disease. J Cell Physiol.

[21] Sarkar D, Leung EY, Baguley BC, Finlay GJ, Askarian-Amiri ME (2015). Epigenetic regulation in human melanoma: past and future. Epigenetics.

[22] Mazar J (2014). The functional characterization of long noncoding RNA SPRY4-IT1 in human melanoma cells. Oncotarget.

[23] Khaitan D (2011). The melanoma-upregulated long noncoding RNA SPRY4-IT1 modulates apoptosis and invasion. Cancer Res.

[24] Luo M (2013). Long non-coding RNA H19 increases bladder cancer metastasis by associating with EZH2 and inhibiting E-cadherin expression. Cancer Lett.

[25] Lin N (2019). Long noncoding RNA MALAT1 potentiates growth and inhibits senescence by antagonizing ABI3BP in gallbladder cancer cells. J Exp Clin Cancer Res.

[26] Gutschner T (2013). The noncoding RNA MALAT1 is a critical regulator of the metastasis phenotype of lung cancer cells. Cancer Res.

[27] Tian Y, Zhang X, Hao Y, Fang Z, He Y (2014). Potential roles of abnormally expressed long noncoding RNA UCA1 and Malat-1 in metastasis of melanoma. Melanoma Res.

[28] Wang X (2014). Long non-coding RNA urothelial carcinoma associated 1 induces cell replication by inhibiting BRG1 in 5637 cells. Oncol Rep.

[29] Su S (2015). Long non-coding RNA BANCR regulates growth and metastasis and is associated with poor prognosis in retinoblastoma. Tumour Biol.

[30] Shi Y (2015). Downregulated Long Noncoding RNA BANCR Promotes the Proliferation of Colorectal Cancer Cells via Downregualtion of p21 Expression. PLoS One.

[31] Flockhart RJ (2012). BRAFV600E remodels the melanocyte transcriptome and induces BANCR to regulate melanoma cell migration. Genome Res.

[32] Liu S, Zhang W, Liu K, Liu Y (2019). LncRNA SNHG16 promotes tumor growth of pancreatic cancer by targeting miR-218-5p. Biomed Pharmacother.

[33] Peng P (2019). Decreased miR-218-5p Levels as a Serum Biomarker in Bone Metastasis of Prostate Cancer. Oncol Res Treat.

[34] Li XC, Hai JJ, Tan YJ, Yue QF, Liu LJ (2019). MiR-218 suppresses metastasis and invasion of endometrial cancer via negatively regulating ADD2. Eur Rev Med Pharmacol Sci.

[35] Hu, X., Ding, D., Zhang, J. & Cui, J. Knockdown of lncRNA HOTAIR sensitizes breast cancer cells to ionizing radiation through activating miR-218. *Biosci Rep* **39**, 10.1042/BSR20181038 (2019). 10.1042/BSR20181038 PMC6449517 30429228

[36] Zhang L (2019). Long noncoding RNA LINC-PINT inhibits non-small cell lung cancer progression through sponging miR-218-5p/PDCD4. Artif Cells Nanomed Biotechnol.

[37] Kardooni Hoda, Gonzalez-Gualda Estela, Stylianakis Emmanouil, Saffaran Sina, Waxman Jonathan, Kypta Robert (2018). CRISPR-Mediated Reactivation of DKK3 Expression Attenuates TGF-β Signaling in Prostate Cancer. Cancers.

[38] Xia P, Xu XY (2017). DKK3 attenuates the cytotoxic effect of natural killer cells on CD133(+) gastric cancer cells. Mol Carcinog.

[39] Mohammadpour H, Pourfathollah AA, Nikougoftar Zarif M, Khalili S (2016). Key role of Dkk3 protein in inhibition of cancer cell proliferation: An in silico identification. J Theor Biol.

