# Author Correction: LINC00459 sponging miR-218 to elevate DKK3 inhibits proliferation and invasion in melanoma

**DOI:** 10.1038/s41598-021-01245-y

**Published:** 2021-11-10

**Authors:** Yuhua Yang, Wenxian Xu, Zhuojun Zheng, Zhihai Cao

**Affiliations:** 1 Department of Dermatology, The Third Affiliated Hospital of Soochow University, Changzhou, China; 2 Department of Pathology, The First Affiliated Hospital of Soochow University, Suzhou, China; 3 Department of Hematology, The Third Affiliated Hospital of Soochow University, Changzhou, China; 4 Department of Emergency, The Third Affiliated Hospital of Soochow University, Changzhou, China

Correction to: *Scientific Reports* 10.1038/s41598-019-55701-x, published online 16 December 2019

The original version of this Article contained errors in Figure 4.

The images presented in Figure 4F are incorrect, as data for Figure 2F were inadvertently included in place of the correct data.

The original Figure 4 and accompanying legend appears below.

**Figure 4:**
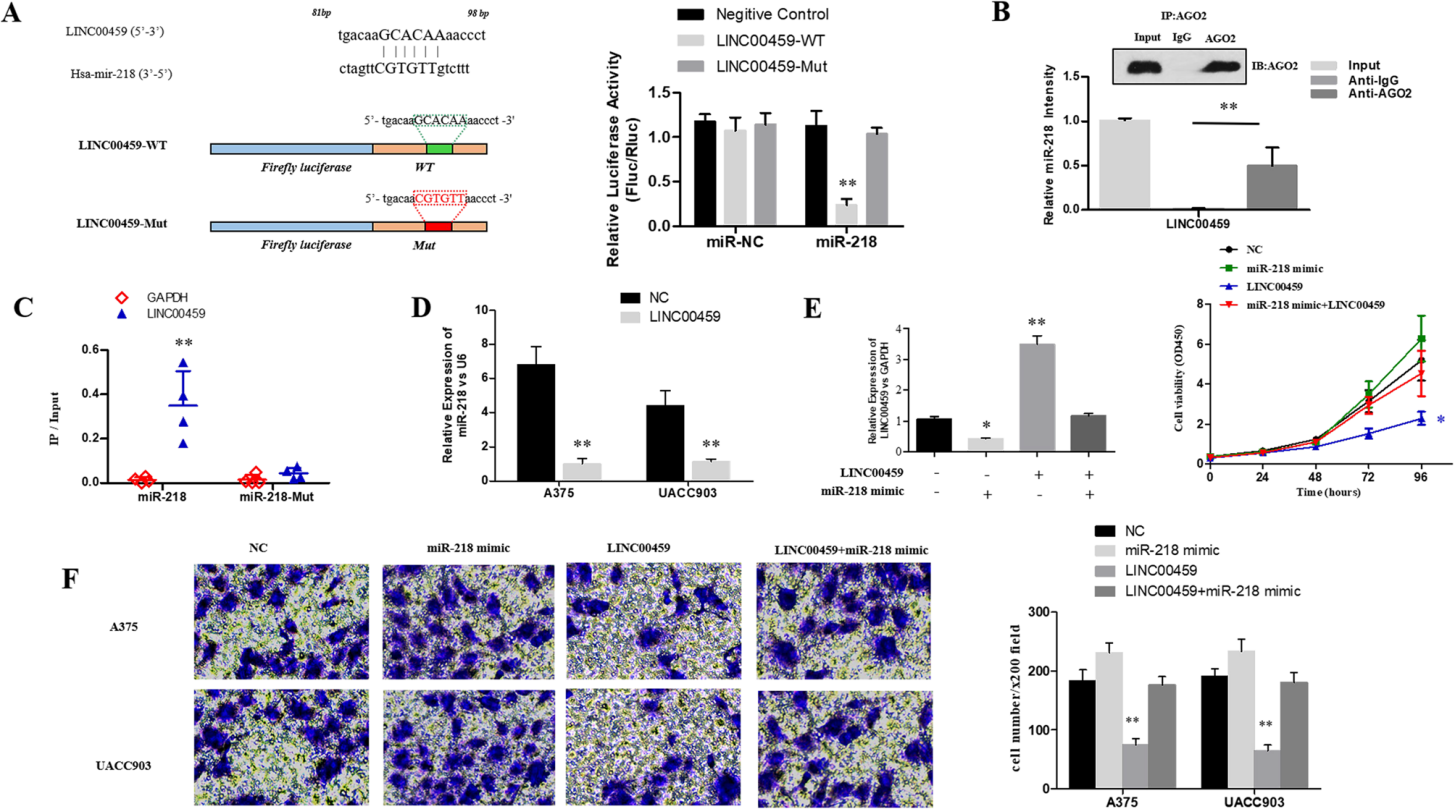
The targeted regulation of the LINC00459 on miR-218. (**A**) The upper diagram showed the schematic figure of the predictive miR-218 binding sites of the LINC00459, and the lower diagram presented the sequences of LINC00459 and LINC00459. The histogram presented the luciferase activities in A375 cells (**P < 0.01); (**B**) Anti-AGO2 RIP assays showed that LINC00459 and miR-218 were more abundant in anti-AGO2 comparing with the anti-IgG immunoprecipitates (**P < 0.01); (**C**) The scatter plot showed that the content of LINC00459 was higher in the miR-218-wt than in miR-218-mut with mutant binding sites of LINC00459 (**P < 0.01); (**D**) The expression of miR-218 in melanoma cells with overexpressed LINC00459 relative to the cells with overexpressed-NC were presented; (**E**) The expression of LINC00459 and cell proliferation after transfection of NC, miR-218 mimic Lv-LINC00459 and miR-218 mimic + Lv-LINC00459 were evaluated by RT-qPCR and CCK-8 assay (*P < 0.05, **P < 0.01); (**F**) The invasion of melanoma cells after transfection of NC, miR-218 mimic, Lv-LINC00459 and miR-218 mimic + Lv-LINC00459 were detected by transwell assay (*P < 0.05, **P < 0.01).

The original Article has been corrected.

