# Supplementary material for: LINC00459 sponging miR-218 to elevate DKK3 inhibits proliferation and invasion in melanoma
Source: Sci Rep. 2019 Dec 16;9:19139. doi: 10.1038/s41598-019-55701-x (PMC6914790; doi:10.1038/s41598-019-55701-x)
Supplement: Supplementary file 1 — Supplementary information [file 41598_2019_55701_MOESM1_ESM.docx]

**LINC00459 sponging miR-218 to elevate DKK3 inhibits proliferation and invasion in melanoma**

Yuhua Yang^1^, Wenxian Xu^2^, Zhuojun Zheng^3^, Zhihai Cao^4^

^1^ Department of Dermatology, The Third Affiliated Hospital of Soochow University, China

^2^ Department of Pathology, The First Affiliated Hospital of Soochow University, China

^3^ Department of Hematology, The Third Affiliated Hospital of Soochow University, China

^4^ Department of Emergency, The Third Affiliated Hospital of Soochow University, China

**Suppl Table 1 Primers used in this study**

| ID | Sequences |
| --- | --- |
| LINC00459 forward | 5’- TGACAAGCACAAAACCCTGC -3’ |
| LINC00459 reverse | 5’- ACTGCTACGCTGCTCTTCTG -3’ |
| DKK3 forward | 5’- GCGCCTCTGATCGCGT-3’ |
| DKK3 reverse | 5’- AAGGTCGGCTTGCACACATA-3’ |
| VOPP1 forward | 5’- GAAGGCGGTGTCATATGTGC-3’ |
| VOPP1 reverse | 5’- ACGAAGACAGAAAGGCCAGG-3’ |
| SGCZ forward | 5’- TTCACTGTGGATAGTCCGCT -3’ |
| SGCZ reverse | 5’- CGGTTAACTGCCCCATGTGA -3’ |
| TPD52 forward | 5’- GGGCCATATTGCAGAACCCT-3’ |
| TPD52 reverse | 5’- GGACTGGGTCTGTTCTCAGC-3’ |
| SAMD12 forward | 5’- AGCATGACATAACTGGGCGA-3’ |
| SAMD12 reverse | 5’- GCAGTGCCATGGGTTAGTCT-3’ |
| ONECUT2 forward | 5’- CATTTAGTCCAAGCCCCGGT-3’ |
| ONECUT2 reverse | 5’- CCGCTCTCATGCAGAGGTAG-3’ |
| SERP1 forward | 5’- CACATTCCCGTTGTTGCGTT-3’ |
| SERP1 reverse | 5’- TTTCTCGAGGTCTTGGCGAC-3’ |
| HECTD2 forward | 5’- ATCCGAAATGAAGGCCCCAG-3’ |
| HECTD2 reverse | 5’- TGGGACAATCTTGAATACTGTTTTT-3’ |
| GAPDH forward | 5’-TGAACGGGAAGCTCACTGG-3’ |
| GAPDH reverse | 5’-TCCACCACCCTGTTGCTGTA-3’ |
| U6 forward | 5’-CTCGCTTCGGCAGCACA-3’ |
| U6 reverse | 5’-AACGCTTCACGAATTTGCGT-3’ |

**Supplemental Figure 1**


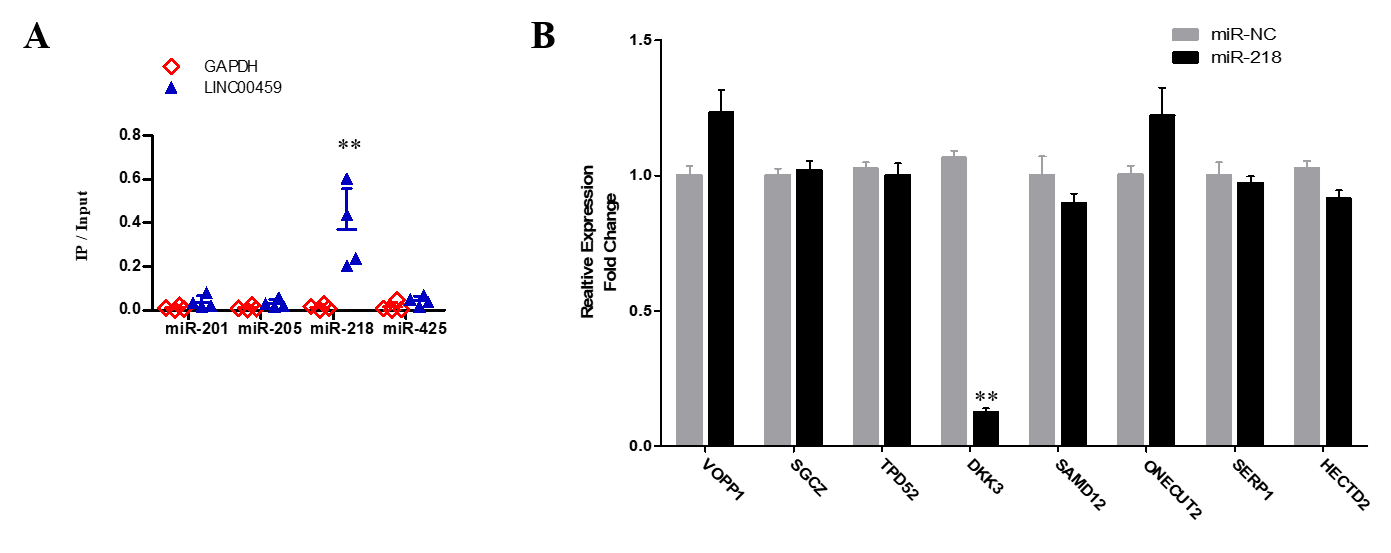


**Supplemental Figure 1: A** Biotinylated miRNA were transfected into A375 cells. RT-qPCR was performed to quantify the RNA levels of LINC00459 and GAPDH. A scatter plot showing the relative ratios of the input of IP. ***P* < 0.01. **A** miR-218 and miR-NC were transfected into A375 cells. RT-qPCR was performed to quantify the RNA levels of several candidates. ***P<*0.01.
